# Expression and preliminary characterization of human MICU2

**DOI:** 10.1242/bio.018572

**Published:** 2016-06-22

**Authors:** Dan Li, Wenping Wu, Hairun Pei, Qiang Wei, Qingzhan Yang, Jimin Zheng, Zongchao Jia

**Affiliations:** 1 College of Chemistry, Beijing Normal University, Beijing 100875, China; 2 Department of Biochemical and Molecular Science, Queen University, Kingston, Ontario, CanadaK7L 3N6

**Keywords:** MICU1, MICU2, Calcium, EF-hand, Mitochondria

## Abstract

MICU2 has been reported to interact with MICU1 and participate in the regulation of mitochondrial Ca^2+^ uptake, although the molecular determinants underlying the function of MICU2 is unknown. In order to characterize MICU2 we screened a series of N-terminal and C-terminal truncations and obtained constructs which can be expressed in abundance, giving rise to soluble samples to enable subsequent characterizations. Size exclusion chromatography (SEC) and multi-angle laser light scattering (MALLS) revealed that MICU2 exists as a monomer in Ca^2+^-free conditions but forms a dimer in Ca^2+^-bound conditions. Unlike MICU1, the C-helix domain of MICU2 exhibits no influence on protein conformation in both Ca^2+^-free and Ca^2+^-bound forms. Furthermore, mutation of the first EF-hand abolishes the ability of MICU2 to switch to a dimer in the presence of Ca^2+^, indicating that the first EF-hand is not only involved in Ca^2+^ binding but also in conformational change. Our pull-down and co-immunoprecipitation assays suggest that, in addition to disulfide bonds, salt bridges also contribute to MICU1-MICU2 heterodimer formation.

## Abstract (precis)

**Summary:** Ca^2+^ induces a monomer-dimer switch of MICU2, with the associated conformational change governed by its first EF-hand, but not influenced by the C-helix domain. A disulfide bond and salt bridge each contribute to MICU1-MICU2 heterodimer formation.

## INTRODUCTION

More than half a century ago it was proposed that Ca^2+^ could accumulate in mitochondria through the mitochondrial calcium uniporter (DeLuca and Engstrom, 1961; Vasington and Murphy, 1962). Subsequent research demonstrated that Ca^2+^ flux across the inner mitochondrial membrane (IMM) was driven by the membrane potential (ΔΨ) which was generated by the respiratory chain (Nicholls, 2005; O'Rourke, 2007; Santo-Domingo and Demaurex, 2010; Drago et al., 2011). However, the molecular identities of the uniporter complex were ambiguous until recently. By screening IMM proteins using selective siRNA, MICU1 was discovered, which heralded the molecular identification of the uniporter complex (Perocchi et al., 2010). Other members of the uniporter complex family (MCU, MCUb, MICU2, EMRE and MCUR1) were identified subsequently (Marchi and Pinton, 2014). A series of evidence has defined MCU as the pore-forming unit of the uniporter channel (Baughman et al., 2011; De Stefani et al., 2011; Chaudhuri et al., 2013; Kovacs-Bogdan et al., 2014). MCUb is a paralog of MCU, considered to be an endogenous dominant negative isoform since its overexpression reduces the number of channels in the lipid bilayer (Raffaello et al., 2013). MICU2, a paralog of MICU1, interacts with MICU1 to form a heterodimer that regulates the function of MCU (Plovanich et al., 2013; Kamer and Mootha, 2014; Patron et al., 2014). Discovered by mass spectrometry of the MCU proteome, EMRE has been found essential for the interaction of MICU1 and MICU2 with MCU (Sancak et al., 2013). MCUR1 was considered to be a key component of the uniporter complex since its knockdown was observed to abolish the activity of the uniporter (Mallilankaraman et al., 2013). Although the molecular identities of the uniporter complex have been revealed, precise understanding of the mitochondrial Ca^2+^ uptake machinery is still largely lacking.

As a regulator that may interact with the pore-forming unit MCU, the MICU family has attracted more attention and as a result, more is now known about it. However, various experimental results from different groups on the effect of MCU-mediate Ca^2+^ uptake caused by MICU1 knockdown are in conflict. Earlier studies found that knockdown of MICU1 inhibited the mitochondrial Ca^2+^ uptake (Perocchi et al., 2010) which was confirmed by subsequent experiments (Alam et al., 2012), whereas other studies discovered that MICU1 knockdown led to constitutive mitochondrial Ca^2+^ (Ca^2+^_m_) accumulation at low intracellular Ca^2+^ concentration (Ca^2+^_i_) (Mallilankaraman et al., 2012; Patron et al., 2014; Csordas et al., 2013; de la Fuente et al., 2014). Thus, MICU1 is assumed to act as the threshold for Ca^2+^ uptake and function to inhibit uniporter-mediated Ca^2+^ uptake at low Ca^2+^_m_ (Mallilankaraman et al., 2012; Csordas et al., 2013). In MICU1, the EF-hands act as a Ca^2+^ sensor, although there are still some controversies about its role. Some studies showed that the EF-hand mutation failed to inhibit Ca^2+^ flux into mitochondrial which paralleled MICU1 knockdown results (Mallilankaraman et al., 2012). In contrast, others revealed that MICU1 with non-function EF-hands still inhibited Ca^2+^ uptake (Csordas et al., 2013; Kamer and Mootha, 2014). Moreover, crystal structures of human MICU1 demonstrated that Ca^2+^ binding by the EF-hand could cause large conformational changes in the interface of the dimer unit (Wang et al., 2014). As a paralog of MICU1, MICU2 shares 27% protein sequence identity and contains two putative EF-hands. It has been shown that MICU2 interacts with MICU1 in a heterodimer through a disulfide bond (Patron et al., 2014; Petrungaro et al., 2015). MICU2 was found to be essential for the MCU-mediated Ca^2+^ uptake, since both its knockout and EF-hand mutation caused reduced Ca^2+^ uptake at high Ca^2+^_i_, even if the effect was smaller than that of MICU1 (Plovanich et al., 2013; Kamer and Mootha, 2014). Nevertheless, other studies found that MICU2 knockdown could enhance Ca^2+^ uptake at high Ca^2+^_i_, suggesting that it acts as the gatekeeper while MICU1 acts as the activator for the uniporter (Patron et al., 2014). A recent report proposed that MICU2 is the inhibitor of MCU and MICU1 has a double role, acting as an inhibitor at low Ca^2+^_i_ and activator at high Ca^2+^_i_ (>2.5 µM) (Matesanz-Isabel et al., 2016). In addition, there are studies pointed out that the MICU1-MICU2 heterodimer binds to MCU in order to close the channel at low Ca^2+^_i_. With the increase of Ca^2+^ concentration, EF-hands of the MICU1-MICU2 heterodimer would bind to Ca^2+^ and result in the release of the dimer from the uniporter (Petrungaro et al., 2015).

Herein, we report the preparation and the characterization of MICU2 using insect and *Escherichia coli* cells. We have constructed and expressed a series of recombinant MICU2 with N-terminal and C-terminal truncations to achieve protein expression, enabling the characterization of MICU2 in Ca^2+^-free and Ca^2+^-bound states using SEC and MALLS. Through site-specific mutation of the EF-hands, we have studied Ca^2+^ response of MICU2, which demonstrates a significant difference with MICU1. Furthermore, we have studied MICU1-MICU2 association and revealed new interaction features.

## RESULTS

### Cloning of full-length MICU2 and truncations

**Fig. 1.**
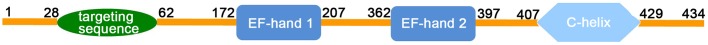
**A schematic drawing of the functional domain of human MICU2.** Like MICU1, MICU2 also possesses a targeting sequence at its N-terminus, two putative EF-hands and a helix domain at the C-terminus.

As shown in Fig. 1, MICU2 possesses an N-terminal targeting sequence, two putative EF-hands and a helix domain at the C-terminus. We designed a series of constructs including full-length and truncated MICU2 with varying lengths. Several constructs of N-terminal deletions were prepared starting at amino acid 66, 84, 105 or 159. The C-helix has been proven crucial in MICU1 (Kamer and Mootha, 2014; Wang et al., 2014), and in order to exploit the role of the C-helix domain in MICU2, the C-terminal clones were constructed by deleting 28 or 36 amino acids in the C-terminus. The details of MICU2 constructs are listed in Table 1. For the pull-down assay of MICU1 and MICU2, MICU1-NΔ54 and MICU1-NΔ96 were cloned without any tag. Furthermore, MICU1-NΔ96 and MICU2-NΔ84 were cloned into the multiple cloning sites (MCS) of the pETDuet-1 vector respectively, which would allow expression of the two constructs simultaneously. Clones of MICU1 (MICU1-NΔ96-Myc) and MICU2 (MICU2-NΔ84-Flag and MICU2-NΔ84-CΔ36-Flag) constructs used for co-immunoprecipitation were designed with Myc-tag and Flag-tag, respectively. The details of MICU1 constructs are listed in Table S1.

**Table 1.**
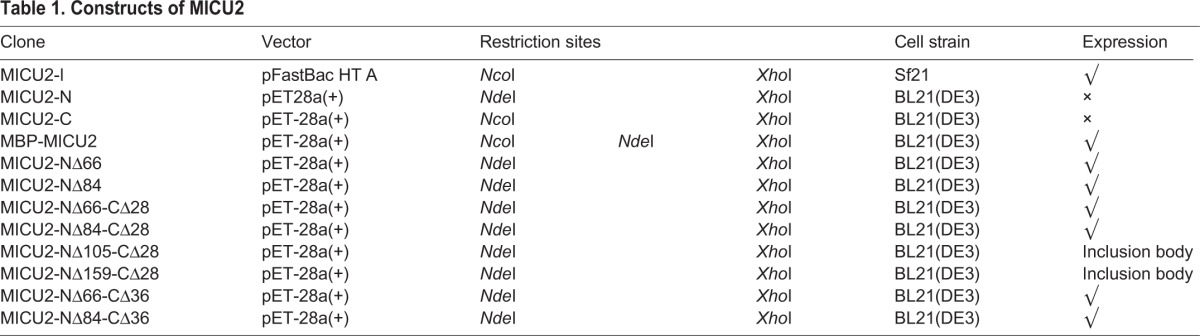
**Constructs of MICU2**

### Protein expression and purification

**Fig. 2.**
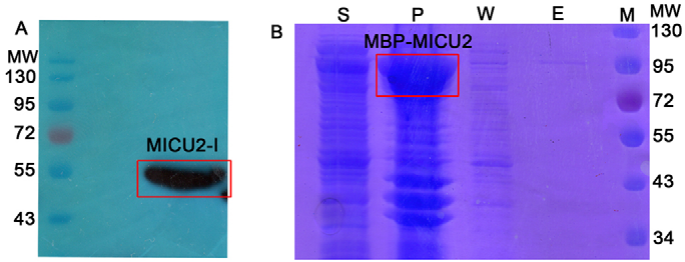
**Expression and purification of full-length MICU2.** (A) MICU2-I expression in Sf21 insect cells and total cell lysate analyzed by western blot. (B) SDS-PAGE showing MBP-MICU2 (∼95 kDa) in the precipitant. (Lane S and P: the supernatant and precipitant samples of MBP-MICU2 after sonication; Lane W: samples from the buffer wash; Lane E: the eluted samples form the resin; Lane M: molecular weight marker).

Our expression results of the MICU2 constructs are summarized in Table 1. For full length constructs, MICU2-I was expressed in Sf21 insect cells. MICU2-N (N-terminal His-tag), MICU2-C (C-terminal His-tag) and maltose-binding protein (MBP)-MICU2 were expressed in *E. coli* BL21 (DE3) cells. Though we were able to detect MICU2-I expression in the insect cells by western blotting (Fig. 2A), the SDS-PAGE results indicated that most of the protein was accumulated in the precipitant, which was further confirmed by western blot (Fig. S1). When fused with a MBP tag, MICU2 was also largely expressed in the precipitant in *E. coli* (Fig. 2B); therefore, we concluded that the constructs with the targeting sequence were all insoluble, even in the presence of a MBP tag. After truncating the targeting sequence, we obtained soluble MICU2-NΔ66 and MICU2-NΔ84 which were purified by affinity chromatography (Fig. S2A,B), however large amounts of the proteins were still present in the precipitant (Fig. S2A,B). We next screened the C-terminal truncations. Several constructs (MICU2-NΔ66-CΔ28, MICU2-NΔ66-CΔ36, MICU2-NΔ84-CΔ28 and MICU2-NΔ84-CΔ36) had good protein expression with high purity (Fig. S2C-F). However, for those constructs with more N-terminal amino acids eliminated, such as MICU2-NΔ105-CΔ28 and MICU2-NΔ159-CΔ28, the protein would express in inclusion bodies in *E. coli*.

### Protein purification by SEC

**Fig. 3.**
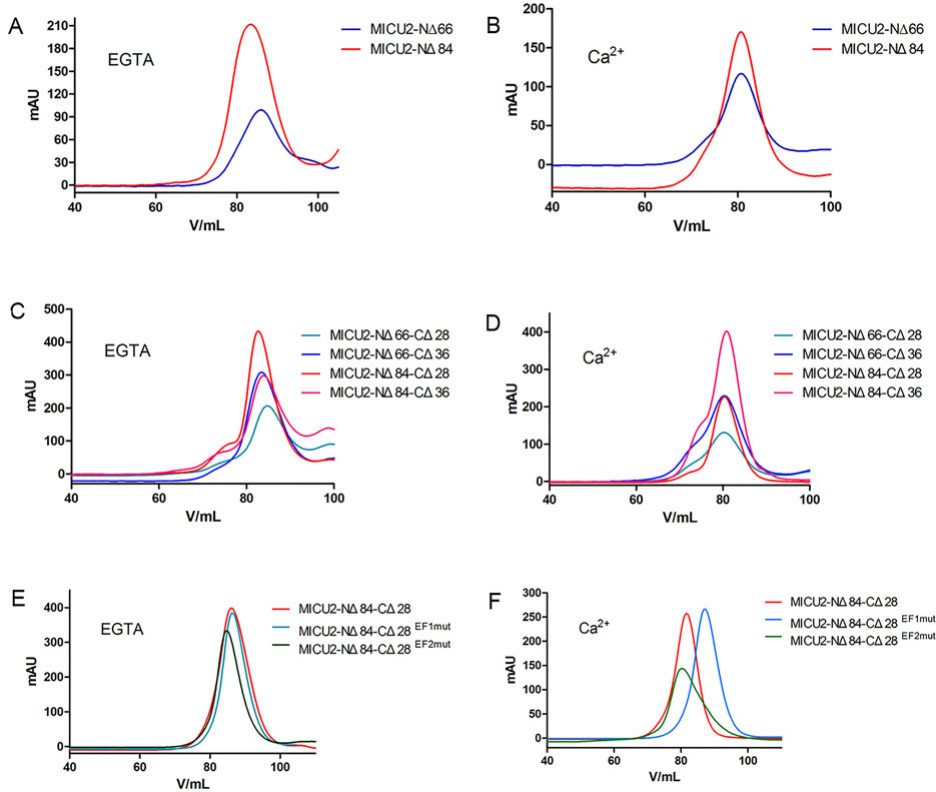
**SEC profiles of MICU2 truncations and EF-hand mutations.** (A,B) SEC results of N-terminal truncations of MICU2 in EGTA or Ca^2 +^conditions. (C,D) SEC results of N-terminal and C-terminal truncations of MICU2 in EGTA or Ca^2+^conditions. (E,F) SEC results of MICU2-NΔ84-CΔ28 and EF-hand mutations in EGTA or Ca^2+^conditions. The V_R_ values for MICU2 terminal truncations were consistent with its C-terminal constructions (comparing A and C or B and D). However, after binding Ca^2+^, there was a 3-5 ml shift of the V_R_ values suggesting that there may be conformational changes which were not influenced by the C-helix (comparing A and B or C and D). SEC for the EF-hand mutants revealed that MICU2-NΔ84-CΔ28^EF2mut^ have V_R_ difference with Ca^2+^ which is not the case for MICU2-NΔ84-CΔ28^EF1mut^, indicating conformational changes were caused by the first EF-hand.

**Fig. 4.**
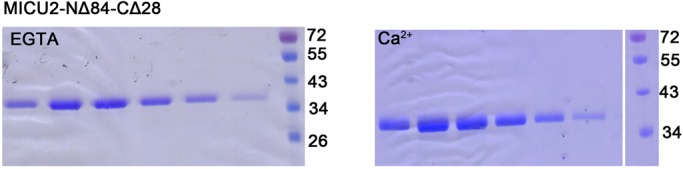
**Fraction analysis of MICU2-NΔ84-CΔ28 in Ca^2+^-free or Ca^2+^-bound conditions.** SDS-PAGE analysis of SEC fractions revealed that MICU2-NΔ84-CΔ28 exhibited the best purity among all the MICU2 truncations. The white vertical line refers to the fact that MW markers were loaded on the same SDS-PAGE, but not in the adjacent lanes.

The eluted samples were concentrated and purified by SEC. Considering the EF-hands of MICU2, the buffer used for SEC either contained EGTA to provide a Ca^2+^-free condition, or CaCl_2_ to maintain the protein in a Ca^2+^-bound state. The results are shown in Fig. 3 and the retention volume (V_R_) values of the peaks are shown in Table S2. In the Ca^2+^-free condition, peak integration revealed that the V_R_ values of MICU2-NΔ66 and MICU2-NΔ84 were ∼83-85 ml (Fig. 3A). Similarly, for the other constructs (MICU2-NΔ66-CΔ28, MICU2-NΔ66-CΔ36, MICU2-NΔ84-CΔ28, MICU2-NΔ84-CΔ36), the V_R_ values were ∼84 ml (Fig. 3C). Therefore, it seems that the C-helix domain has no significant influence on the protein conformation in Ca^2+^-free condition. In Ca^2+^-bound state, the V_R_ values were ∼80 ml for all aforementioned six MICU2 constructs, indicating that the C-helix domain has no influence on the protein conformation in the Ca^2+^-binding state as well (Fig. 3B,D). However, comparing the V_R_ values of all tested constructs between EGTA and Ca^2+^ conditions, consistently there was a 3-5 ml shift which is not insignificant, suggesting that there may be conformational changes after binding Ca^2+^. SDS-PAGE results of various constructs are shown in Fig. 4, Figs S2 and S3, in which the construct MICU2-NΔ84-CΔ28 exhibited the best purity.

To further explore the EF-hand of MICU2, we prepared two mutants (MICU2-NΔ84-CΔ28^EF1mut^ and MICU2-NΔ84-CΔ28^EF2mut^) by mutating the key residues of the Ca^2+^-binding loop of each EF-hand. The SEC analysis results of the mutants are shown in Fig. 3E,F and the V_R_ values are given in Table S3. We were able to obtain a better shape of the elution peak, which represents more homogeneous protein, after carrying out SEC purification twice. In the Ca^2+^-free condition the V_R_ of MICU2-NΔ84-CΔ28 was almost the same with its EF-hand mutants (85-86 ml), however the V_R_ for MICU2-NΔ84-CΔ28 and MICU2-NΔ84-CΔ28^EF2mut^ shifted to 80-81 ml when Ca^2+^ was added, while for MICU2-NΔ84-CΔ28^EF1mut^ it was still 87 ml. Thus, in the Ca^2+^-bound condition, MICU2-NΔ84-CΔ28 and MICU2-NΔ84-CΔ28^EF2mut^ have a different conformation which is not the case for MICU2-NΔ84-CΔ28^EF1mut^.

### Multi-angle laser light scattering (MALLS)

**Fig. 5.**
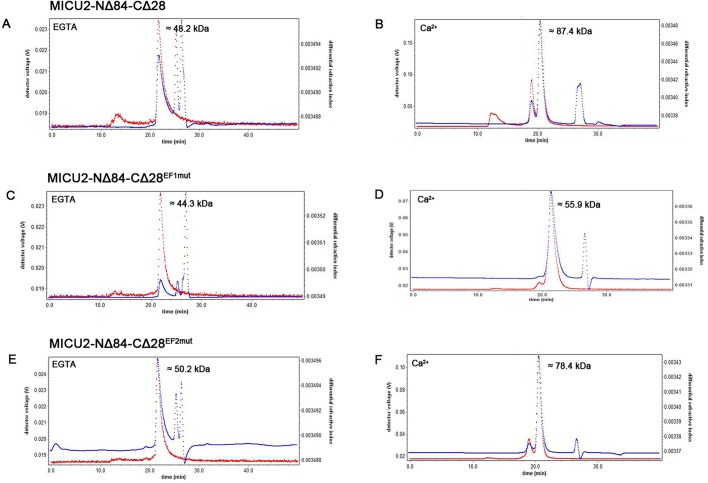
**MALLS results for MICU2-NΔ84-CΔ28 and EF-hand mutations.** (A,C,E) MALLS results for MICU2-NΔ84-CΔ28 and EF-hand mutations in Ca^2+^-free (2 mM EGTA) condition. (B,D,F) MALLS results for MICU2-NΔ84-CΔ28 and EF-hand mutations in Ca^2+^-bound (5 mM CaCl_2_) condition. Ca^2+^ could change MICU2-NΔ84-CΔ28 and MICU2-NΔ84-CΔ28^EF2mut^ from a monomer to a dimer which is not the case for MICU2-NΔ84-CΔ28^EF1mut^. Therefore, the conformational change would take place in parallel with Ca^2+^ binding with the first EF-hand. The red line indicates the laser light scattering data and the blue line indicates the differential refractive index data.

To further investigate the conformation of MICU2 in the Ca^2+^-free and Ca^2+^-bound states, we performed MALLS analysis of MICU2-NΔ84-CΔ28, MICU2-NΔ84-CΔ28^EF1mut^ and MICU2-NΔ84-CΔ28^EF2mut^ (Fig. 5). Similar molecular weights for MICU2-NΔ84-CΔ28 and EF-hand mutations were detected in the Ca^2+^-free condition, as the MALLS results were 48.2 kDa, 44.3 kDa and 50.2 kDa, respectively. However, when Ca^2+^ was added, the MALLS results of MICU2-NΔ84-CΔ28 and EF-hand mutants were 87.4 kDa, 55.9 kDa and 78.4 kDa, respectively. The predicted molecular weight of MICU2-NΔ84-CΔ28 is ∼40 kDa. Considering the influence of protein purity, it is not surprising that there may be a deviation between the experimental result and the actual molecular weight. However, our MALLS results are sufficiently clear that MICU2-NΔ84-CΔ28 and MICU2-NΔ84-CΔ28^EF2mut^ existed as a monomer without Ca^2+^ and switched to a dimer after binding Ca^2+^. Interestingly, MICU2-NΔ84-CΔ28^EF1mut^ did not undergo the similar monomer-dimer transition and behaved only as a monomer. This somewhat surprising result suggests that MICU2-NΔ84-CΔ28^EF1mut^ would not be influenced by Ca^2+^, and the conformational change is controlled by the first EF-hand. Moreover, our SEC results in Fig. 3 show that the addition of Ca^2+^ would cause a retention volume shift for MICU2-NΔ84-CΔ28 and MICU2-NΔ84-CΔ28^EF2mut^ which did not happen for MICU2-NΔ84-CΔ28^EF1mut^. These results are consistent with the MALLS analysis.

### Pull-down and co-immunoprecipitation assays of MICU1 and MICU2

**Fig. 6.**
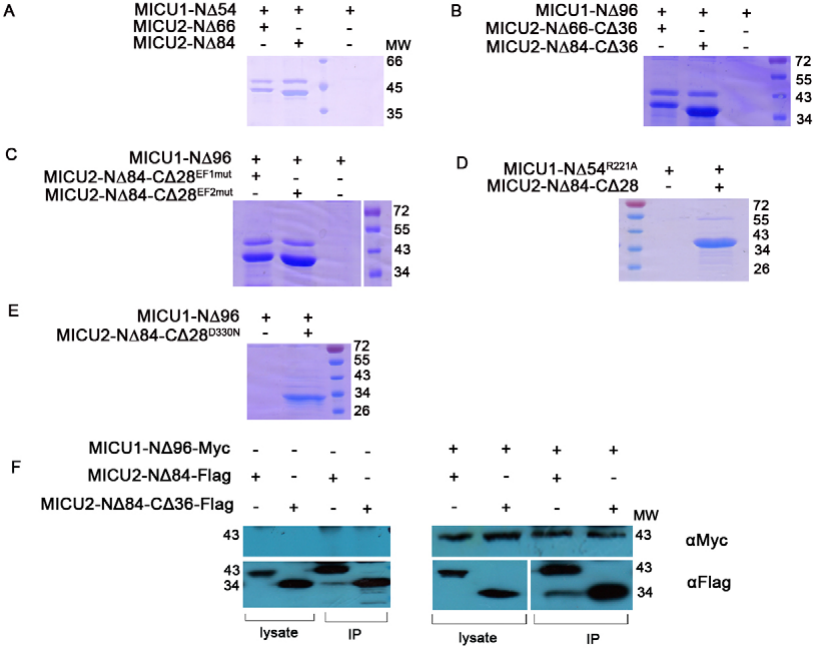
**Pull-down analysis using reducing SDS-PAGE.** (A) As expected, MICU1-NΔ54 interacts with MICU2-NΔ66 and MICU2-NΔ84 which contain Cys413. (B) In the absence of Cys413, MICU1-NΔ96 still interacts with MICU2-NΔ66-CΔ36 and MICU2-NΔ84-CΔ36. (C) The EF-hands of MICU2 are not involved in its interaction with MICU1. (D,E) In addition to the disulfide bond, salt bridge formed by Arg221 in MICU1 and Asp330 in MICU2 contributes to the MICU1-MICU2 interaction. (F) Western blot analysis of co-immunoprecipitation of MICU1-Δ96-Myc with MICU2-NΔ84-Flag and MICU2-NΔ84-CΔ28-Flag reveals the interaction both in the presence and absence of Cys413, consistent with the *in vitro* pull-down results (A,B). The white vertical line refers to the fact that MW markers were loaded on the same SDS-PAGE, but not in the adjacent lanes.

It has been reported that MICU1 and MICU2 form a heterodimer via a disulfide bond through the cysteine residues in the C-helix domain (Cys463 in MICU1 and Cys413 in MICU2) (Patron et al., 2014; Petrungaro et al., 2015). In order to characterize this bonding, we performed *in vitro* pull-down assay and the results are shown in Fig. 6 and Fig. S5. In this experiment, we first confirmed the disulfide bond between MICU1 and MICU2 through the co-expression experiment (Fig. S5). The western blot shows that the two constructs could form a complex with a molecular weight ∼95 kDa no matter if the His-tag is contained in MICU1-NΔ96 or MICU2-NΔ84. In order to explore whether there are other types of interaction between the heterodimer, MICU1 truncations were designed without any tag while the constructs for MICU2 have His-tags in the N-terminus. As a result, both MICU1 constructs and mutations would not bind to the Ni-NTA resin which were confirmed by SDS-PAGE (the control groups are blank in Fig. 6). For both MICU1 (MICU1-NΔ54) and MICU2 (MICU2-NΔ66 and MICU2-NΔ84) in which the C-helix domain is intact, there are two bands in each experimental group which are readily visualized (Fig. 6A), clearly indicating MICU1-MICU2 interaction as anticipated. However, for the constructs of MICU2-NΔ66-CΔ36 and MICU2-NΔ84-CΔ36 which lack Cys413 and would therefore not be expected to form the heterodimer, there are still two bands (Fig. 6B). This surprising result demonstrates that both MICU2-NΔ66-CΔ36 and MICU2-NΔ84-CΔ36 retain interaction with MICU1-NΔ96. To further substantiate these unexpected observations, we carried out co-immunoprecipitation and western blot analysis, which clearly indicates that MICU2 (MICU2-NΔ84-Flag and MICU2-NΔ84-CΔ36-Flag) interacts with MICU1-Δ96-Myc either with or without Cys413 (Fig. 6E). Thus, it seems that there are other factors contributing to MICU1-MICU2 interaction in addition to the disulfide bond. In order to determine whether the EF-hands are involved in the interaction of the heterodimer, pull-down assays were next performed using MICU1-NΔ96 and MICU2 EF-hand mutants. Interestingly, both the two MICU2 EF-hand mutants which lack Cys413 and Ca^2+^ binding residues still interacted with MICU1, indicating that the EF-hands do not participate in MICU1-MICU2 interaction (Fig. 6C). According to the structure model of MICU1 (Wang et al., 2014), we designed the mutants MICU1-NΔ54^R221A^ and MICU2-NΔ84-CΔ28^D330N^. Neither mutants interacted with the corresponding wild-type MICU2-NΔ84-CΔ28 or MICU1-NΔ96 as there was only one band present (Fig. 6D,E). Therefore, MICU1 associates with MICU2 through the salt bridge created by Arg221 in MICU1 and Asp330 in MICU2, in addition to the disulfide bond.

## DISCUSSION

In order to gain further functional insights, it is necessary to establish a reliable method of producing recombinant MICU2 for in-depth characterization. Our experiments show that constructs containing an N-terminal targeting sequence did not express in *E. coli*. Subsequently, we attempted the insect expression system and MBP fusion construct. Although the expression was successful, unfortunately the protein was mostly insoluble. Lacking the N-terminal target sequence, constructs MICU2-NΔ66 and MICU2-NΔ84 improved both protein solubility and expression level (Figs S1 and S2). The SEC results revealed that there was significant shift between the retention volume for MICU2-NΔ66 and MICU2-NΔ84 in Ca^2+^-free and Ca^2+^-bound condition, indicating that there was conformational change induced by Ca^2+^ binding.

The helix domain of MICU1 in the C-terminus is considered to be essential for protein conformation. It is reported that in the wild-type MICU1 C-helices bundle together to form Ca^2+^-free hexamer, while the C-helix truncation behaves as a dimer in both Ca^2+^-free and Ca^2+^-bound conditions (Wang et al., 2014). Considering the similarity of the functional domain between MICU1 and MICU2, we wondered whether the C-helix domain would play a similarly important role in MICU2 as well. Our results revealed that when the C-terminal 28 or 36 amino acids were truncated, the constructs exhibited similar properties as the N-terminal constructs in either Ca^2+^-free or Ca^2+^-bound condition. Thus, the C-helix in MICU2 seems to have no influence on protein conformation as observed for MICU1. The MALLS results further revealed that Ca^2+^ triggers MICU2-NΔ84-CΔ28 to switch from a monomer to a dimer. Therefore, the conformational change of MICU2 is largely controlled through the combined effect of EF-hands and Ca^2+^ binding. A recent paper reports that MICU1 would rearrange in a disaggregated form (Waldeck-Weiermair et al., 2015) while our results reveal that MICU2 aggregates switch from monomer to dimer after binding Ca^2+^. This difference may be related to their functional role in mitochondrial Ca^2+^ uptake as MICU1 and MICU2 exert opposite effects on MCU activity (Patron et al., 2014; Matesanz-Isabel et al., 2016). Therefore, conformational change of MICU1 and MICU2 after binding Ca^2+^ may be able to disable MICU2's inhibitory effect and evoke MICU1's activating function for mitochondrial Ca^2+^ uptake.

To discern the influence of each EF-hand on conformational change, we probed the two EF-hands by site-directed mutagenesis. It has been reported that EF-hand mutants of MICU1 with a C-terminus truncation behave as dimers and would not be influenced by Ca^2+^ (Wang et al., 2014). Unexpectedly, in our SEC experiment, Ca^2+^ caused a retention volume shift for MICU2-NΔ84-CΔ28 and MICU2-NΔ84-CΔ28^EF2mut^ but not for MICU2-NΔ84-CΔ28^EF1mut^. In order to substantiate these observations, we carried out further MALLS experiments and the results revealed that MICU2-NΔ84-CΔ28^EF2mut^ changed from a monomer to a dimer after binding Ca^2+^ while MICU2-NΔ84-CΔ28^EF1mut^ did not. Therefore, it is the first EF-hand, not the second, which induces the conformational change upon Ca^2+^ binding.

**Fig. 7.**
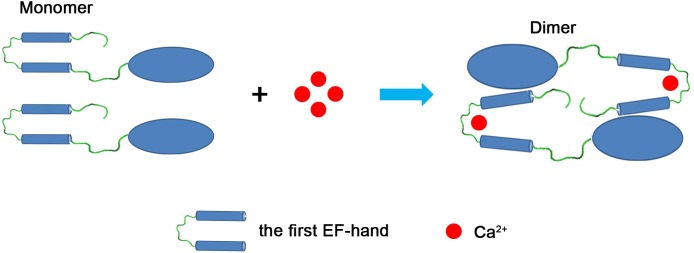
**Model showing conformational change of MICU2 and its switch from monomer to dimer.** In the Ca^2+^-free condition, MICU2 is a monomer. The binding of Ca^2+^ causes conformational change of the first EF-hand which triggers its interaction with the other MICU2 monomer.

Our observed conformational change of MICU2 caused by the first EF-hand is different from that of MICU1. Ca^2+^-free structure of MICU1 suggested that the dimer interaction is via a salt bridge between Asp376 and Arg221. Upon binding Ca^2+^, hydrogen bonds between Arg221 and Glu224 from one monomer with Ser382 and His385 from another, which provide the interaction between dimers (Wang et al., 2014). Since MICU2 structure has not been determined, our conjecture of the conformation change of MICU2 is described as follows (Fig. 7). Alignment of MICU1 and MICU2 shows that only the aspartic acid is conserved (Asp376 in MICU1 and Asp330 in MICU2). The corresponding residue of MICU1 Arg221 in MICU2 is Ser175; the salt bridge would not form for MICU2 and this may be the reason why MICU2 is a monomer without Ca^2+^. The switch of MICU2 from monomer to dimer induced by Ca^2+^ binding in the first EF-hand may be considerably different from MICU1 since all the residues contributing to MICU1 dimer formation are not conserved in MICU2. The first EF-hand may experience large conformational change after binding Ca^2+^ and result in potential new interactions between the first EF-hand and the other MICU2 monomer.

Previous experiments indicate that MICU1 and MICU2 form a heterodimer through disulfide bonds between Cys463 in MICU1 and Cys413 in MICU2. MICU1 can also form a homodimer when it is overexpressed, which is consistent with its crystal structures (Patron et al., 2014; Petrungaro et al., 2015). By using non-reducing SDS-PAGE analysis, we confirmed that there is a disulfide bond between MICU1 and MICU2. However, our pull-down results using constructs lacking Cys413 suggest that the disulfide bond is not the only interaction between MICU1 and MICU2 which is further confirmed by co-immunoprecipitation *in situ*. Since the aspartic acid is conserved (Asp376 in MICU1 and Asp330 in MICU2), based on the structural analysis we speculate that in the heterodimer, Arg221 in MICU1 may interact with Asp330 in MICU2 to form a salt bridge similar to the Asp376-Arg221 salt bridge in the MICU1 homodimer. Pull-down results using Arg221 or Asp330 mutants provide strong support for our hypothesis.

### Conclusion

Through construct screening by truncating the N-terminus or C-terminus of MICU2, we have successfully obtained soluble MICU2 truncations which enabled subsequent characterization. The SEC and MALLS experiments demonstrate that MICU2 exists as a monomer without Ca^2+^ and changes to a dimer after binding Ca^2+^. Mutation of the first EF-hand, but not the second, abolishes the Ca^2+^-induced conformational change of MICU2. Compared with MICU1, our results indicate a different role of the first EF-hand in MICU2 which binds Ca^2+^ and induces conformational change. In addition to the disulfide bond, our results suggest a salt bridge also contributes to the MICU1-MICU2 interaction. A full understating of the molecular determinants underlying these new observations would have to wait for structural characterization.

## MATERIALS AND METHODS

### Cloning of MICU1 and MICU2

The details of various clones for MICU1 and MICU2 are given in Table 1 and Table S1. Generally, human MICU1 (BC004190.2) or MICU2 (BC031089.1) genes were amplified by PCR with the desired restriction sites and cleaved with the corresponding restriction enzymes (Thermo Scientific). The digested PCR products were ligated into vectors, namely pFastBac HT A for insect expression, pET-28a(+) and pETDuet-1 for *E. coli* expression, and pcDNA3.1 for HEK293T expression, respectively. It should be mentioned that for the construction of MBP-MICU2, MBP tag was first cloned into pET-28a(+) between the *Nco*I and *Nde*I restriction sites (a Prescission protease cleavage site was inserted before the *Nde*I restriction site). Next the cleaved MICU2 PCR products (5′ *Nde*I, 3′ *Xho*I) were ligated into the modified vector which was digested using the same enzymes. For MICU1, the reverse primers were designed with a termination codon to ensure lacking of His-tag. For MICU2, clones were designed with His-tag in the N-terminus. In order to co-express MICU1 and MICU2, we introduced both genes into the pETDuet-1 vector which contains two MCSs. Therefore, the first protein inserted into the first MCS contains His-tag and the second protein does not have any tag. After that, the recombinant vectors were transformed into TOP10 cells and cultured on the agar LB medium plate overnight at 37°C. A single clone was picked up, cultivated and confirmed by DNA sequencing. The recombinant plasmid was extracted (Plasmid DNA Miniprep Kit, Tiangen) and stored at −40°C for further use.

### Site-specific mutation of MICU2 constructs

To disable Ca^2+^ binding, mutation of the EF-hand was performed according to literature by mutating the first and the last residues of the predicted 12 residues in Ca^2+^ binding loop of each EF-hand to alanine and to lysine, respectively (Perocchi et al., 2010). To investigate the interaction of MICU1 and MICU2, we also designed MICU1-R221A and MICU2-D330N mutants. The mutation was operated by PCR using KOD polymerase (TOYOBO). After purification by agarose gel electrophoresis and DNA purification kit (Tiangen), the PCR product was incubated with *Dpn*I for 30 min. The digested product was next transformed into TOP10 and cultured on the agar LB medium plate overnight. A single clone was picked and cultured and the mutated sites were confirmed by DNA sequencing.

### Protein expression

The expression of the full-length MICU2 in insect cells was done using the Bac-to-Bac baculovirus expression system (Invitrogen). The recombinant donor plasmid containing MICU2 gene was first transformed into competent DH10Bac *E. coli* cells and incubated on the agar LB medium plate (50 µg/ml kanamycin, 7 µg/ml gentamicin, 10 µg/ml tetracycline, 100 µg/ml X-gal and 40 µg/ml IPTG) for 48 h at 37°C. A white clone was picked and cultured in 15 ml LB medium (50 µg/ml kanamycin, 7 µg/ml gentamicin, 10 µg/ml tetracycline) overnight. The recombinant bacmid was isolated (Plasmid Mini Kit, Qiagen) and analyzed by PCR using pUC/M13 primers (pUC/M13 forward: 5′-CCCAGTCACGACGTTGTAAAACG-3′; pUC/M13 reverse: 5′-AGCGGATAACAATTTCACACAGG-3′) to confirm that the target gene was inserted into the bacmid. Purified recombinant bacmid (∼1 µg) was used to transfect Sf21 insect cells (∼1×10^6^ cells) for 3 days using the Cellfectin reagent (Cellfectin II reagent, Invitrogen) to produce P1 viral stock. The high-titer P2 viral stock was generated by using the P1 viral stock to transfect the Sf21 insect cells (∼1.2×10^6^ cells in 25 ml medium). P3 viral stock was generated using the P2 viral stock with the same method. To express the protein, Sf21 cells (1 litre) were first suspension cultured to a density of 1×10^6^ to 2×10^6^ cells/ml and infected with the P3 viral stock (20 ml) for 48 h before the cells were harvested. The expression of MICU2 was determined by western blotting (anti-His-tag mouse monoclonal antibody, CWbiotech, Cat No. CW0286).

For the expression of MICU1, MICU2 and the coexpression of MICU1-MICU2 construct in *E. coli*, the recombinant plasmids were transformed into BL21 (DE3) *E. coli* strain. A single clone was picked and cultured in 5 ml LB medium for 12 h, and 1 ml cells were then transferred into 100 ml LB medium to amplify overnight. 30 ml of the amplified cells were inoculated into 1 litre TB medium (100 µg/ml kanamycin) and cultivated (37°C, 150 rpm) until the OD_600_ reached ∼0.8. 0.5 mM Isopropyl β-D-1-thiogalactopyranoside (IPTG) was added after the system cooled to 16°C and the cells were continued to cultivate for 20 h at 16°C. Cells were harvested by centrifugation and stored at −40°C until further use.

### Protein purification

The general protocol for the purification of the full-length and truncated MICU2 is as follows. The harvested cells were first re-suspended in buffer I (20 mM Tris pH 7.0, 300 mM NaCl, 20 mM imidazole, 0.3% Triton X-100). For the insect cells, cells were lysed by freezing and thawing. For *E. coli* cells, cells were lysed by sonication. Both the cell lysates were centrifuged at 18,000 rpm (39,000× ***g***) for 30 min. The column with 2 ml of Ni-NTA resin (Qiagen) was first equilibrated with buffer I and precooled at 4°C and the supernatant was slowly loaded onto the resin. Afterwards, the protein was washed with 150-200 ml buffer II (20 mM Tris pH 7.0, 300 mM NaCl, 50 mM imidazole) and eluted with 20 ml buffer III (20 mM Tris pH 7.0, 300 mM NaCl, 400 mM imidazole). Protein purity in each step was monitored by 12% SDS-PAGE. The eluted protein was concentrated by a 10-kDa molecular weight cut-off centrifugal filter (Merck Millipore) and samples were further analyzed by SEC using a 120 ml Hiload Superdex 200 10/300 GL column (GE Healthcare Biosciences AB) on AKTA purifier system. The column was equilibrated with buffer IV (20 mM MES pH 6.8, 300 mM NaCl, 2 mM EGTA or 5 mM CaCl_2_) before protein analysis. The protein was detected by a UV detector at a wavelength of 280 nm and the sample of each fraction was assessed by 12% SDS-PAGE.

### Multi-angle laser light scattering

The molecular weight range and polydispersity of MICU2-NΔ84-CΔ28 and its EF-hand mutants were analyzed by SEC equipped with a MALLS detector. Protein samples were prepared by concentrating to 0.5-2.5 mg/ml and filtered through a 0.22-μm filter. 200 μl of the protein sample was injected into the loop and chromatographic analysis was done using KW-G and KW-803 columns (Shimadzu) at a flow rate of 0.5 ml/min. The protein signals were detected by a DAWN HELEOS-II light scattering detector and an Optilab rex refractive index detector (Wyatt Technology). The molecular weight was calculated by peaks integration (ASTRA software version 5.3.4.13). Each sample was carried out independently and repeated at least three times.

### Pull-down assay for MICU1 and MICU2

Pull-down assays for MICU1 and MICU2 were operated by co-expression or by mixing cells individually expressing MICU1 or MICU2 together. The harvested cells of MICU1 constructs (MICU1-NΔ54, MICU1-NΔ54^R221A^, MICU1-NΔ96) with MICU2 constructs (MICU2-NΔ66-CΔ28, MICU2-NΔ66-CΔ36, MICU2-NΔ84-CΔ28, MICU2-NΔ84-CΔ28, MICU2-NΔ84-CΔ36, MICU2-NΔ84-CΔ28^EF1mut^, MICU2-NΔ84-CΔ28^EF2mut^, MICU2-NΔ84-CΔ28^D330N^) were re-suspended in buffer I, and the mixed cells were sonicated together. Cell lysates were centrifuged and the supernatant was applied to the Ni-NTA resin that was precooled and equilibrated with the buffer I. The resin was washed with 100 ml buffer II and the proteins were eluted together with buffer III. The controls for the pull-down assay were performed by purifying MICU1-NΔ54, MICU1-NΔ96 and MICU1-NΔ54^R221A^ as aforementioned individually. All eluted samples were analyzed by reducing SDS-PAGE. The co-expression cells (MICU1-NΔ96-MICU2-NΔ84 or MICU2-NΔ84-MICU1-NΔ96) were purified by the protocols aforementioned. The eluted samples were exposed to air for oxidation and ensuring disulfide formation and then analyzed by western blot with non-reducing SDS-PAGE. The assays were carried out independently and repeated at least three times.

### Transfection and co-immunoprecipitation of MICU1 and MICU2

HEK293T cells were cultured in Dulbecco's modified Eagle's medium (DMEM, Hyclone GE Healthcare Life Sciences) supplemented with 10% v/v fetal bovine serum (FBS, Gibco) and 1% v/v penicillin-streptomycin (PS, Caission). The cells were cultured in a 95% air and 5% CO_2_ environment at 37°C. MICU1-NΔ96-Myc with MICU2-NΔ84-Flag or MICU2-NΔ84-CΔ36-Flag genes were transfected into cells using Lipofectamine 3000 (Invitrogen, Cat No. 1756121) and cultured sequentially in a 10-cm Petri dish for 48 h. Transfected HEK293T cells were harvested by PBS buffer (Hyclone, GE Healthcare Life Sciences) and then lysed in 300 µl of TBS buffer (20 mM Tris pH 7.4, 150 mM NaCl) containing 0.2% n-dodectl-β-D-maltoside (DDM, Sigma) and protease inhibitor cocktail (Roche). After being lysed on ice for 30 min, the lysate was centrifuged at 13,000× ***g*** for 30 min and 40 µl of the cell supernatant was removed and used as positive control. Protein A/G agarose beads (GE Healthcare) were washed three times by TBS buffer and then incubated with anti-Flag antibody (Transgen, Cat No. HT201) and cell supernatant for 4 h. The beads were washed three times by TBS and eluted by adding 50 µl of SDS-PAGE loading buffer. Both the control and eluted samples were analyzed by western blotting using anti-Flag antibody and anti-c-Myc antibody (Transgen, Cat No. HT101).
